# RELIGIOSITY AND HEALTH AMONG CHINESE OLDER ADULTS IN MAINLAND CHINA, HONG KONG, AND TAIWAN: A META-ANALYTIC REVIEW

**DOI:** 10.1093/geroni/igz038.1932

**Published:** 2019-11-08

**Authors:** Jia Li, Qi Wang

**Affiliations:** 1 The Hong Kong Polytechnic University, Hong Kong, Hong Kong; 2 University of Hong Kong, Hong Kong, Hong Kong

## Abstract

Abstract: Religion plays an important role in people’s later life. However, most existing studies on health and religiosity focused on Western settings. China has the largest aging population in the world and distinct contexts of religion. This study aims to examine the relationship between religiosity and health of Chinese older adults through a meta-analysis. We conducted a comprehensive database (English and Chinese) and gray literature searching. Two researchers independently extracted the studies and evaluated the quality of the eligible ones. A random-effect model was adopted to combine the results. Hedges’ g was computed as a standardized measure of the effect size. Subgroup analysis was conducted to examine the potential moderators. From the 3776 potentially eligible papers, 74 were eventually included. The results showed that, having a religious belief or ever attending religious activities was significantly related to a higher level of anxiety (Hedges’ g= 0.392, 95% CI[0.230, 0.556]), escape acceptance of death (0.477[ 0.154, 0.801]), death avoidance (0.498 [0.127, 0.870]), death anxiety (0.448[0.122, 0.774]), more positive coping practices (0.581[0.073, 1.094]), and subjective social support (0.313[0.143, 0.483]). However, the subgroup analysis did not conclude any significant moderators. Religiosity is significantly related to a variety of psychosocial characteristics of older adults, including both negative and positive traits. It calls for more future studies to investigate the competing mechanisms regarding how religiosity can influence older adults’ health and vice versa.

